# Supplementary material for: Pre-Endoscopic Scores Predicting Low-Risk Patients with Upper Gastrointestinal Bleeding: A Systematic Review and Meta-Analysis
Source: J Clin Med. 2023 Aug 9;12(16):5194. doi: 10.3390/jcm12165194 (PMC10456043; doi:10.3390/jcm12165194)
Supplement: Supplementary file 1 [file jcm-12-05194-s001.zip › jcm-2454731-supplementary.pdf]

Supplementary Table S1

Search Strategy:

----

1 exp stomach/
2 stomach.tw.
3 gastr$.tw.
4 exp duodenum/
5 duoden$.tw.
6 exp esophagus/
7 esophag$.tw.
8 oesophag$.tw.
9 peptic$.tw.
10 or/1-9
11 exp peptic ulcer/
12 exp peptic ulcer hemorrhage/
13 (peptic adj5 ulcer$).tw.
14 (bleed$ adj5 ulcer$).tw.
15 (rebleed$ adj5 ulcer$).tw.
16 (recurrent adj5 bleed$ adj5 ulcer$).tw.
17 (acute adj5 bleed$ adj5 ulcer$).tw.
18 (ulcer$ adj5 hemo$).tw.
19 (ulcer$ adj5 haemo$).tw.
20 nonvaric$.tw.
21 (non adj5 varic$).tw.
22 (gastrointestinal adj5 bleed$).tw.
23 (gastrointestinal adj5 rebleed$).tw.
24 (gastrointestinal adj5 hemorrhag$).tw.
25 (gastrointestinal adj5 haemorrhag$).tw.
26 UGIB.tw.
27 (GI adj25 bleed$).tw.
28 or/11-27
29 10 and 28

30 Blatchford.tw,kw.
31 Rockall.tw,kw.
32 AIMS65.tw.
33 (aims adj1 "65").tw.
34 (Progetto adj1 Nazionale adj1 Emorragia adj1 Digestiva).tw.
35 pned.tw.
36 (early adj1 discharge).tw,kw.
37 prognostic scale*.tw,kw.
38 prognostic score*.tw,kw.
39 (ABC adj1 score).tw.
40 or/30-39
41 29 and 40

Supplementary Table S2 – Primary and secondary outcomes for the extra risk assessment scores (expressed as proportions)

	pBBS			pCSMCPI	HUPS	CANUKA	
	**0**	**≤1**	**≤2**	**0**	**0**	**0**	**≤1**
**Composite outcome**	NA	NA	NA	NA	0.29 (0.15-0.45)<br><br>I^2=45%<br>Studies=2<br>N=1,309	NA	NA
**Mortality**	0.00 (0.00-0.15)<br><br>I^2=NA<br>Studies=1<br>N=831	0.00 (0.00-0.09)<br><br>I^2=NA<br>Studies=1<br>N=831	0.00 (0.00-0.05)<br><br>I^2=0%<br>Studies=1<br>N=831	0.00 (0.00-0.08)<br><br>I^2=NA<br>Studies=1<br>N=831	0.00 (0.00-0.35)<br><br>I^2=NA<br>Studies=1<br>N=831	0.00 (0.00-0.34)<br><br>I^2=NA<br>Studies=1<br>N=1606	0.00 (0.00-0.03)<br><br>I^2=NA<br>Studies=1<br>N=1606
**Rebleeding**	NA	NA	NA	NA	0.00 (0.00-0.35)<br><br>I^2=NA<br>Studies=1<br>N=831	0.11 (0.00-0.48)<br><br>I^2=NA<br>Studies=1<br>N=1606	0.03 (0.01-0.08)<br><br>I^2=NA<br>Studies=1<br>N=1606
**Blood transfusion**	NA	NA	NA	NA	NA	0.00 (0.00-0.34)<br><br>I^2=NA<br>Studies=1<br>N=1606	0.00 (0.00-0.03)<br><br>I^2=NA<br>Studies=1<br>N=1606
**Endoscopic intervention**	0.00 (0.00-0.15)<br><br>I^2=NA<br>Studies=1<br>N=831	0.01 (0.00-0.12)<br><br>I^2=NA<br>Studies=1<br>N=831	0.06 (0.2-0.08)<br><br>I^2=NA<br>Studies=1<br>N=831	0.09 (0.05-0.18)<br><br>I^2=NA<br>Studies=1<br>N=831	NA	0.11 (0.00-0.48)<br><br>I^2=NA<br>Studies=1<br>N=1606	0.01 (0.00-0.05)<br><br>I^2=NA<br>Studies=1<br>N=1606
**Surgical intervention**	NA	NA	NA	NA	0.00 (0.00-0.35)<br><br>I^2=NA<br>Studies=1<br>N=831	0.00 (0.00-0.34)<br><br>I^2=NA<br>Studies=1<br>N=1606	0.00 (0.00-0.03)<br><br>I^2=NA<br>Studies=1<br>N=1606
**Radiological intervention**	NA	NA	NA	NA	0.00 (0.00-0.35)<br><br>I^2=NA<br>Studies=1<br>N=831	0.00 (0.00-0.34)<br><br>I^2=NA<br>Studies=1<br>N=1606	0.00 (0.00-0.03)<br><br>I^2=NA<br>Studies=1<br>N=1606

Results expressed as proportions (95% confidence interval)

NA: Not available

Supplementary Table S3 – The Newcastle-Ottawa scale for the quality of included studies

		Selection				Comparability	Outcome			
**Criteria**	**Design**	**Representativeness of exposed cohort**	**Selection of controls**	**Ascertainment of exposure**	**Demonstration that outcome of interest was not present at the start of study**	**Study controls for baseline imbalances**	**Assessment of outcome**	**Was follow up long enough for outcome to occur**	**Adequacy of follow up of cohort**	**Score**
Acceptable (*)		Patient receiving a risk assessment using a validated pre-endoscopic tool for upper gastrointestinal bleed	Similar to exposed cohort but high-risk for poor outcomes	Secured records or prospective allocation	Outcomes assessed after presenting with upper gastrointestinal bleeding	Yes or Not applicable	Secured records or blinded prospective assessment	At least 30 days follow up	Less than 10% loss to follow up	
Ak et al.	R	*	*	*	*	NA	*	*	*	7
Aquarius et al.	P	*	*	*	*	NA	*	*	*	7
Brainster et al.	R	*	*	*	*	NA	*	*	*	7
Bryant et al.	P	*	*	*	*	NA	*	-	*	6
Chan et al.	R	*	*	*	*	NA	*	*	*	7
Chatten et al.	R	*	*	*	*	NA	*	*	*	7
Girardin et al.	P	*	*	*	*	NA	*	*	*	7
Gralnek et al.	R	*	*	*	*	NA	*	*	*	7
Jansen et al.	R	*	*	*	*	NA	*	-	-	5
Johnston et al.	R	*	*	*	*	NA	*	*	*	7
Jimenez-Rosales et al.	R	*	*	*	*	NA	*	*	*	7
Kayah et al.	R	*	*	*	*	NA	*	-	*	6
Kherad et al.	R	*	*	*	*	NA	*	*	*	7
Lahiff et al.	R	*	*	*	*	NA	*	*	*	7
Laursen et al. 2012	P	*	*	*	*	NA	*	*	*	7
Laursen et al. 2014	P	*	*	*	*	NA	*	*	*	7
Leiman et al.	R	*	*	*	*	NA	*	-	-	5
Lima et al.	P	*	*	*	*	NA	*	*	*	7
Lu et al.	R	*	*	*	*	NA	*	-	*	6
Matsuhashi et al.	R	*	*	*	*	NA	*	-	*	6
Meltzer et al.	R	*	*	*	*	NA	*	-	*	6
Mustafa et al.	P	*	*	*	*	NA	*	*	*	7
Oakland et al.	R	*	*	*	*	NA	*	*	-	6
Pang et al.	P	*	*	*	*	NA	*	*	*	7
Park et al.	R	*	*	*	*	NA	*	-	*	6
Robins et al.	R	*	*	*	*	NA	*	*	*	7
Ryan et al.	R	*	*	*	*	NA	*	*	*	7
Samreen et al.	R	*	*	*	*	NA	*	-	*	6
Sasaki et al.	R	*	*	*	*	NA	*	*	*	7

Schiefer et al.	R	*	*	*	*	NA	*	*	*	7
Shresthat et al.	P	*	*	*	*	NA	*	*	*	7
Stanley et al. 2009	P	*	*	*	*	NA	*	*	*	7
Stanley et al. 2017	P	*	*	*	*	NA	*	*	*	7
Stephens et al.	P	*	*	*	*	NA	*	*	*	7
Tham et al.	R	*	*	*	*	NA	*	-	*	6
Thanapirom et al.	P	*	*	*	*	NA	*	-	*	6
Yaka et al.	P	*	*	*	*	NA	*	*	*	7
Zhong et al.	P	*	*	*	*	NA	*	-	*	6

NA: Not applicable
R: Retrospective
P: Prospective

Supplementary Table S4 – Sensitivity analyses for primary outcome

	Number of studies	Number of patients	OR (95% CI)	Heterogeneity
Year of publication				
Rockall score 0				
Pre 2012	-	-	-	-
2012-2023	1	388	0.17 (0.08;0.34)	-
Higher quality	1	388	0.17 (0.08;0.34)	-
Rockall score ≤ 1				
Pre 2012	-	-	-	-
2012-2023	-	-	-	-
Higher quality	-	-	-	-
Rockall score ≤ 2				
Pre 2012	-	-	-	-
2012-2023	1	478	0.02 (0.00; 0.16)	-
Higher quality	1	478	0.02 (0.00; 0.16)	-
Blatchford score 0				
Pre 2012	-	-	-	-
2012-2023	-	-	-	-
Higher quality	-	-	-	-
Blatchford score ≤ 1				
Pre 2012	-	-	-	-
2012-2023	1	569	0.00 (0.00;0.02)	-
Higher quality	1	569	0.00 (0.00;0.02)	-
Blatchford score ≤ 2				
Pre 2012	-	-	-	-
2012-2023	1	1102	0.01 (0.00;0.04)	0%
Higher quality	1	1102	0.01 (0.00;0.04)	0%
Aim 65 score 0				

Pre 2012	-	-	-	-
2012-2023	-	-	-	-
Higher quality	-	-	-	-
Aim 65 score ≤ 1				
Pre 2012	-	-	-	-
2012-2023	-	-	-	-
Higher quality	-	-	-	-
ABC score ≤ 1				
Pre 2012	-	-	-	-
2012-2023	-	-	-	-
Higher quality	-	-	-	-
ABC score ≤ 3				
Pre 2012	-	-	-	-
2012-2023	1	645	0.42 (0.29; 0.62)	-
Higher quality	1	645	0.42 (0.29; 0.62)	-

Fixed effect model was not conducted as heterogeneity was noted

	Number of studies	Number of patients	% (95%CI)	Heterogeneity
Year of publication				
Rockall score 0				
Pre 2012	-	-	-	-
2012-2023	2	171	0.13 (0.09; 0.19)	0%
Higher quality	3	355	0.19 (0.15; 0.24)	71%
Rockall score ≤ 1				
Pre 2012	-	-	-	-
2012-2023	-	-	-	-
Higher quality	-	-	-	-
Rockall score ≤ 2				
Pre 2012	-	-	-	-
2012-2023	-	-	-	-

Higher quality	-	-	-	-
Blatchford score 0				
Pre 2012	1	228	0.00 (0.00; 0.03)	-
2012-2023	4	119	0.03 (0.01; 0.09)	0%
Higher quality	4	307	0.01 (0.00; 0.04)	0%
Blatchford score ≤ 1				
Pre 2012	2	91	0.05 (0.02;0.12)	0%
2012-2023	1	146	0.00 (0.00; 0.02)	-
Higher quality	3	237	0.04 (0.02; 0.09)	49%
Blatchford score ≤ 2				
Pre 2012	1	107	0.06 (0.02; 0.12)	0%
2012-2023	2	241	0.01 (0.00; 0.04)	0%
Higher quality	2	241	0.01 (0.00; 0.04)	0%
Aim 65 score 0				
Pre 2012	-	-	-	-
2012-2023	-	-	-	-
Higher quality	-	-	-	-
Aim 65 score ≤ 1				
Pre 2012	-	-	-	-
2012-2023	-	-	-	-
Higher quality	-	-	-	-
ABC score ≤ 1				
Pre 2012	-	-	-	-
2012-2023	-	-	-	-
Higher quality	-	-	-	-
ABC score ≤ 3				
Pre 2012	-	-	-	-
2012-2023	-	-	-	-
Higher quality	-	-	-	-
